# Radionuclide ventriculography phase analysis for risk stratification of patients undergoing cardiotoxic cancer therapy

**DOI:** 10.1007/s12350-020-02277-z

**Published:** 2020-08-03

**Authors:** K. A. Jones, A. D. Small, S. Ray, D. J. Hamilton, W. Martin, J. Robinson, N. E. R. Goodfield, C. A. Paterson

**Affiliations:** 1 Department of Nuclear Cardiology, Glasgow Royal Infirmary, Glasgow, UK; 2 School of Physics and Astronomy, University of Glasgow, Glasgow, UK; 3 School of Mathematics & Statistics, University of Glasgow, Glasgow, UK; 4 School of Medicine, Dentistry & Nursing, University of Glasgow, Glasgow, UK

**Keywords:** RNA: planar, dyssynchrony, diagnostic and prognostic application, image analysis

## Abstract

**Background:**

Accurate diagnostic tools to identify patients at risk of cancer therapy-related cardiac dysfunction (CTRCD) are critical. For patients undergoing cardiotoxic cancer therapy, ejection fraction assessment using radionuclide ventriculography (RNVG) is commonly used for serial assessment of left ventricular (LV) function.

**Methods:**

In this retrospective study, approximate entropy (ApEn), synchrony, entropy, and standard deviation from the phase histogram (phase SD) were investigated as potential early markers of LV dysfunction to predict CTRCD. These phase parameters were calculated from the baseline RNVG phase image for 177 breast cancer patients before commencing cardiotoxic therapy.

**Results:**

Of the 177 patients, 11 had a decline in left ventricular ejection fraction (LVEF) of over 10% to an LVEF below 50% after treatment had commenced. This patient group had a significantly higher ApEn at baseline to those who maintained a normal LVEF throughout treatment. Of the parameters investigated, ApEn was superior for predicting the risk of CTRCD. Combining ApEn with the baseline LVEF further improved the discrimination between the groups.

**Conclusions:**

The results suggest that RNVG phase analysis using approximate entropy may aid in the detection of sub-clinical LV contraction abnormalities, not detectable by baseline LVEF measurement, predicting a subsequent decline in LVEF.

**Electronic supplementary material:**

The online version of this article (10.1007/s12350-020-02277-z) contains supplementary material, which is available to authorized users.

## Introduction

Survival from breast cancer has improved substantially over the last 20 to 30 years due to earlier diagnosis and advances in treatment with adjuvant radiotherapy and chemotherapy. However, cardiotoxicity as a result of this therapy is now the leading cause of morbidity and mortality for survivors.^1^,^2^

Radiotherapy and anthracycline/trastuzumab-based chemotherapy regimens have been associated with increased risk of cardiovascular disease.^3^ Anthracycline-based regimens are associated with the dose-dependent risk of Type 1 cardiotoxicity and heart failure, while trastuzumab is generally associated with reversible Type 2 cardiotoxicity. However, permanent cardiac dysfunction can occur with both Type 1 and 2 cardiotoxicity, despite intervention.^4^ The risk of cancer therapy-related cardiac dysfunction (CTRCD) increases significantly when trastuzumab is combined with anthracyclines.^5^ Cardiac monitoring is required for patients receiving anthracycline/trastuzumab-based treatments, and currently, this relies on the serial assessment of left ventricular ejection fraction (LVEF). Each patient will have a baseline LVEF measurement, then serial LVEF assessment every 3 months during treatment. The European Society of Cardiology (ESC) guidelines consider a 10% point decrease of LVEF to below the lower limit of normal (< 50%) to be an indicator of cardiotoxicity and recommend treatment is altered or stopped to prevent further left ventricular (LV) dysfunction or the development of symptomatic heart failure.^6^

One potential limitation of the current guidelines is that LVEF decline is often a late phenomenon. Therefore, it would be useful if we can identify sub-clinical cardiac abnormalities and identify patients who are at higher risk before treatment starts.

Radionuclide ventriculography (RNVG), also commonly known as multi-gated acquisition study (MUGA) or cardiac blood pool imaging, is a well-established technique that can reproducibly measure ejection fraction and is commonly used to assess LV function in patients undergoing cardiotoxic cancer therapy. Echocardiography is also widely used to assess LVEF but is limited by operator variability and poor reproducibility of LVEF measurement, especially in patients post mastectomy who have had reconstructive surgery. LV dyssynchrony can be assessed with a number of imaging techniques. Recently, there has been increased interest in echocardiography deformation assessment with global longitudinal strain (GLS), as an early marker of LV dysfunction for chemotherapy patients.^6^

This work aims to determine if phase parameters applied to baseline RNVG phase images can measure sub-clinical contraction abnormalities prior to treatment to predict which patients are at a higher risk of CTRCD. At present, there are no published studies investigating RNVG phase parameters as a predictor of CTRCD.

### RNVG Phase Images

**Figure 1:**
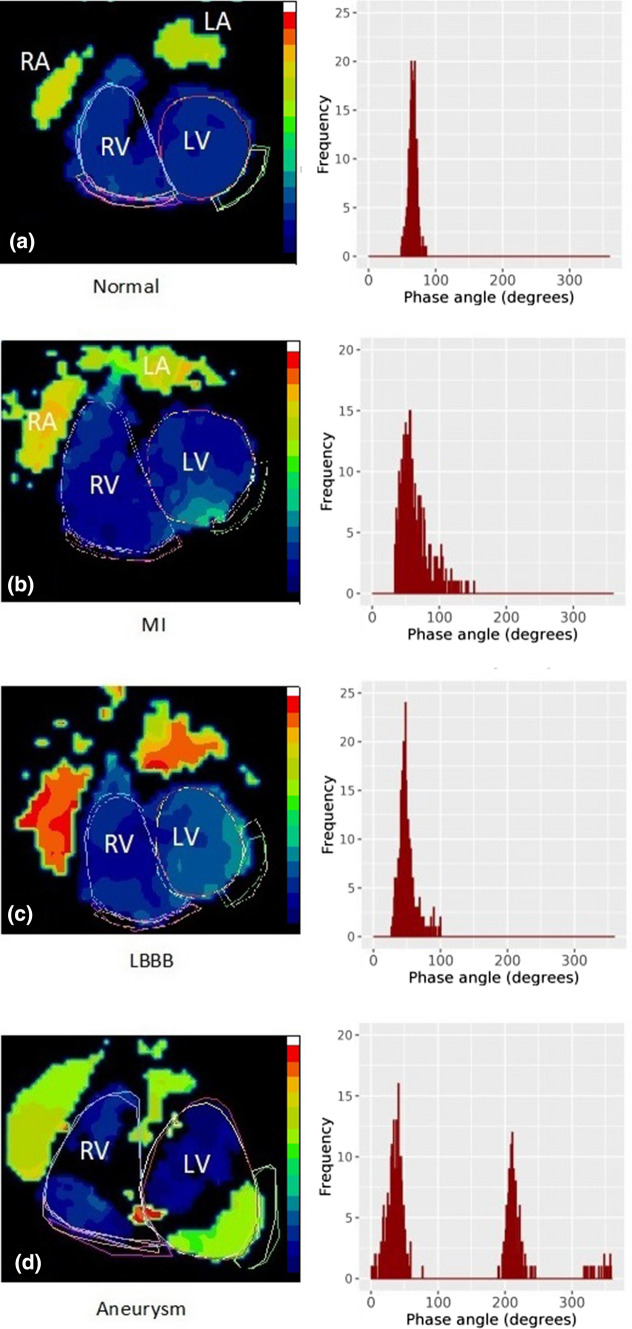
Example images showing phase pattern and associated LV phase histogram for **a** a normal patient with similar phase values throughout the ventricles, **b** an MI patient with late phase values in the area of an apical MI, **c** a patient with left bundle branch block, where there is a gradual change in phase values across the LV, and **d** a patient with a large aneurysm where two distinctly separate segments within the LV are contracting at different times

Phase images, representing the timing of contraction, can be created from RNVG data to provide additional information on ventricular function.^7^,^8^ The timing of contraction, relative to the R wave of the ECG, is obtained from the time-activity curve for each pixel in the RNVG image to create a phase map; a higher phase angle indicates delayed contraction within the region. In a phase image with normal contraction, the pixels within the LV should all be a similar phase value, representing synchronous contraction. Regions of dyssynchronous contraction will appear as delays in the phase images/histograms. This is illustrated in Figure 1, where the phase for a patient with an aneurysm, left bundle branch block (LBBB), or myocardial infarction (MI) has a distinctly different phase pattern compared to a patient with normal contraction. This technique can also detect more subtle phase abnormalities. Phase data from RNVG images can be quantitatively assessed using the mean and the standard deviation of the phase histogram.

### Approximate Entropy

Various measures have been established to quantify dyssynchrony. Most parameters previously investigated are from first-order statistics calculated from the phase histogram such as phase standard deviation (SD). O’Connell et al. derived synchrony to describe the contraction of the left ventricle using the phase and amplitude data extracted from the region of interest, and entropy (from Shannon information measure^9^) as a measure of randomness of contraction within the ventricle.^10^ They demonstrated that synchrony and entropy were superior to phase SD for discriminating between normal and abnormal contraction.

However, parameters based on first-order statistics such as synchrony, entropy, and phase SD do not take into account the spatial relation between the pixel values. More advanced statistical parameters can be used to quantitatively assess ventricular phase.

Approximate entropy (ApEn) is a regularity statistic developed from Kolmogorov–Sinai entropy by Pincus.^11^ When applied to RNVG phase images, ApEn is a statistical measure of dyssynchrony within the ventricle. ApEn calculates the probability that a series of length m remains similar within a tolerance *r* at the next point in the data series, and unlike entropy, takes into account the similarity of adjacent data points and thus can accommodate spatial information more accurately.

ApEn is defined as

$$ {\text{ApEn}} = - \,(N - m)^{ - 1} \mathop \sum \limits_{i = 1}^{N - m} { \ln }\left[ {\frac{{c_{{i^{m + 1} }} \left( r \right)}}{{c_{{i^{m} }} }}} \right], $$

where $N$ is the length of data, $m$ is the sequence length, $r$ is the tolerance, $c_{{i^{m + 1} }} \left( r \right)$ is conditional probability that when a sequence is within the tolerance then the next element will also be within tolerance. The pixel values are considered as a data series. Each group of ‘$m$’ pixels will be compared to every other group of ‘$m$’ pixels within the ROI, including itself. If the group is within the tolerance value $r$, it will be counted. This is carried out for every group of ‘$m$’ pixels then repeated with groups of ‘$m + 1$’ to calculate probabilities $c_{{i^{m} }}$ and $c_{{i^{m + 1} }}$.

ApEn includes a ‘self match’ of vectors creating a bias towards regularity. Several publications also suggest that ApEn lacks relative consistency, meaning that the value of ApEn can ‘flip’ when the input parameters are varied.^12^ For this reason, the input parameters ($m$ and $r$) must be fixed when comparing datasets. At present, there is no established $m$, $r$ or normal range for ApEn applied to RNVG phase images. The values of $m$ and $r$ that are used will markedly affect the results, so it is essential to optimize the input parameters for the data being considered.

ApEn is well established in other fields including gait analysis and heart rate variability but has not previously been widely investigated for assessing ventricular contraction.^13^–^20^ Cullen et al. investigated change in ApEn for serial assessment of 8 patients receiving Herceptin.^21^ This work found a significant change in ejection fraction and ApEn over the course of treatment, however, further work with a larger patient group is necessary. While change in ApEn has been considered, ApEn as a predictive marker has not yet been investigated.

## Method

### ApEn Optimization

Test data were created to simulate patient phase images using in-house code written in R 3.6.3 (R Development Core Team, Vienna, Austria),^22^ allowing *m* and *r* to be tested in a controlled environment. The code allowed the mean and SD in each radial segment to be altered individually, allowing abnormal segments to be introduced. Some publications suggest using a *r* value which is 0.1-0.2 SD of the dataset and *m *= 2,^11^,^23^ although no justification for this choice was found in the literature review. This was used as a starting point to select the test range. A range of *m* between 1 and 5, and *r* between 0.25 and 21 were tested using the simulated data. Patient phase images representing normal, LBBB, and MI were subsequently used to test the results.

**Figure 2:**
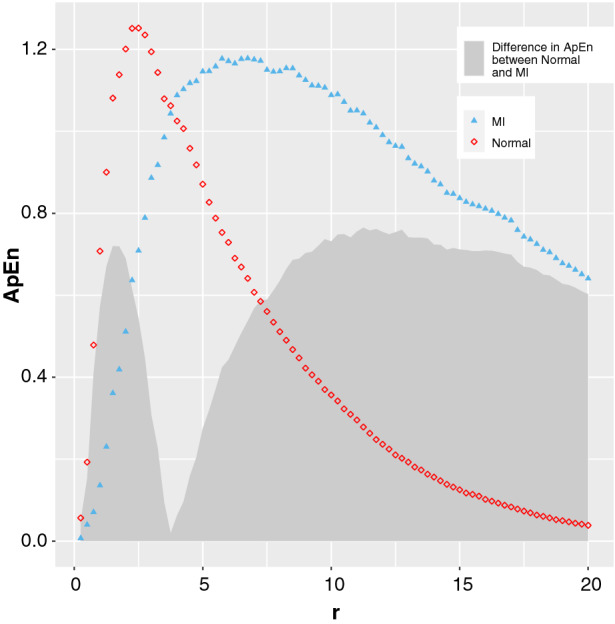
Simulated data representing phase image for a normal LV (in red) and for a large MI (in blue), showing the variation of ApEn with tolerance *r*, where *m* = 2. The shaded area represents the difference in ApEn between the normal and MI phase image in this example

There is a value of *r* where the ApEn calculated from both normal and abnormal phase images is equal, as demonstrated in Figure 2. This plot shows an example of the variation in ApEn values comparing a simulated normal and MI phase image across a range of tolerances *r*. This emphasizes the need for choosing an appropriate value of *r* for the data. If a lower value of *r* is selected then a higher ApEn is normal, while if a larger value of *r* is selected a higher ApEn represents abnormal phase. This is consistent with the work published by Yentes et al.^12^ The most important factor in the selection is avoiding the area where abnormal and normal are equal. This ‘flip’ point, where ApEn cannot discriminate between normal and abnormal, will vary depending on how abnormal the phase is, so the range of flip points for the data type must be considered.

After optimization, the final *m* and *r* values used were *m* = 2, *r* = 7. These values were also tested across the clinical range of left ventricle sizes using simulated data, to ensure consistency with an increase in the number of pixels in the data series, N. The optimization work carried out with both simulated and patient data provides confidence that the selected input values are appropriate.

### Data Acquisition

A retrospective study was undertaken to review 193 consecutive female patients (mean age: 54) who had an RNVG scan at Glasgow Western Infirmary Hospital between 2005 and 2008. All patients included in this study had a baseline RNVG before receiving cardiotoxic cancer therapy. Each patient had serial 24-frame gated RNVG scans, acquired at intervals of approximately 3 months for up to 2 years following the baseline study, with each patient having between 2 and 9 RNVGs. Patients with a baseline LVEF of < 55% were excluded from the study.

In-vivo labeling was performed using intravenous administration of pyrophosphate 20 minutes prior to injection of Technetium-99m pertechnetate. The administered dose for each scan was 800 MBq (21.6 mCi).

Each study was acquired with a Picker 3000XP 3-headed gamma camera (Picker International, Cleveland Heights, Ohio, USA) with a low-energy high-resolution collimator. The gamma camera was positioned to achieve the best septal separation and the scan was acquired for 5 million counts using frame mode acquisition and a matrix size of 64 × 64. The LVEF was assessed by an experienced operator using Picker Lightbox software, with a manual dual region technique to measure the ejection fraction.

For this study only the raw images were available, therefore the baseline study for all 193 patients was reprocessed using MAPS Link medical software. Phase and amplitude images were created from the first-order Fourier harmonic. For each baseline study a single left ventricle region was manually drawn, using the end-diastolic image with reference to the phase and amplitude images. Sixteen patients were excluded at this stage (11 patients with gating problems, which were picked up from the phase image and time-activity curve, 4 patients with a baseline LVEF below 55%, and 1 patient with a baseline scan below diagnostic quality due to poor radiopharmaceutical labeling), leaving 177 patients.

### Data Analysis

Following each patient’s baseline scan, the LVEF from all subsequent studies was compared to the baseline to establish the maximum LVEF drop. The reported LVEF from the original analysis was used, along with the phase images created using MAPS Link Medical software. Based on the ESC guidelines, patients were split by LVEF decline into 2 groups, those who maintained a normal LVEF and those who had an LVEF drop of over 10% to below 50%.

In-house software written in R 3.6.3 was used to calculate ApEn for the baseline scans.^24^ The software creates a data series from the pixels in each region of interest within the phase image, starting from the top left it reads the image from left to right, then right to left on the line below until it reaches the bottom, meaning each group of ‘m’ pixels will be adjacent to each other.

ApEn calculations were carried out on the phase images of the baseline scans, using input parameters *m* = 2 and *r* = 7. Synchrony, entropy, and phase SD were also included for comparison.

### Statistical Analysis

Shapiro–Wilk’s test was used to check the normality of the distribution for each parameter. To test multivariate normality the Henze–Zirkler test was used. Significance testing was performed for each parameter, using the unpaired *t* test or Wilcoxon-signed-rank test, based on the outcome of the univariate test of normality. Hotelling’s *T*^2^ test was used to determine if there was a significant difference between multivariate means of the different populations.^25^

A logistic regression model was fitted in R using all of the phase parameters and the interaction between ApEn and baseline LVEF. A second logistic regression model was created which excluded all of the non-significant variables. A chi-squared test was used to assess the overall significance of the logistic regression models. The area under the receiver operator curve (AUC) and significance was reported for both models.

Random Forest and Naive-Bayes classifiers were fitted using all predictors with the caret package in R,^26^ with 10-fold cross-validation and 3 repeats. The AUC was calculated for each classifier.

A *P* value of < 0.05 was considered significant for all tests. All data analysis and statistics were performed in R 3.6.3.^22^,^27^,^28^

## Results

Patients were split into 2 groups based on the change in LVEF during treatment. Group 1 maintained a normal LVEF (> 50%) during treatment, and Group 2 had a decline in LVEF of more than 10% to below 50%. The guidelines would recommend that the treatment for Group 2 is altered or stopped. There was no significant difference (*P* > 0.05) in age between the two groups.

ApEn, phase SD, and age were normally distributed while synchrony, entropy, and baseline LVEF were not. Multivariate normality testing for ApEn combined with baseline LVEF revealed that both groups were normally distributed.

The results for ApEn, synchrony, entropy, and phase SD are summarized in Table 1. There was a significant difference (*P *< 0.05) in ApEn and LVEF at baseline between the groups. The combination of ApEn and baseline LVEF was also significantly different between the two groups. The other parameters were not significant.

**Table 1:** Summary of results for each phase parameter

	Mean ± SD		Significance test	*P* value
	Group 1 Maintained normal LVEF	Group 2 > 10% drop to LVEF below 50%		
Number of patients	166	11		
Age	55 ± 11	56 ± 15	Wilcoxon-rank-signed	0.799
Synchrony	0.991 ± 0.004	0.989 ± 0.004	Wilcoxon-rank-signed	0.121
Entropy	0.559 ± 0.040	0.584 ± 0.028	Wilcoxon-rank-signed	0.054
ApEn	0.348 ± 0.107	0.418 ± 0.076	Two sample *t* test	0.014
Phase SD	7.90 ± 1.96	8.91 ± 1.78	Wilcoxon-rank-signed	0.094
Baseline LVEF	73.5 ± 6.1	64.5 ± 6.7	Two sample *t* test	< 0.001
(ApEn, baseline LVEF)			Hotelling’s *T*^2^	< 0.001

**Figure 3:**
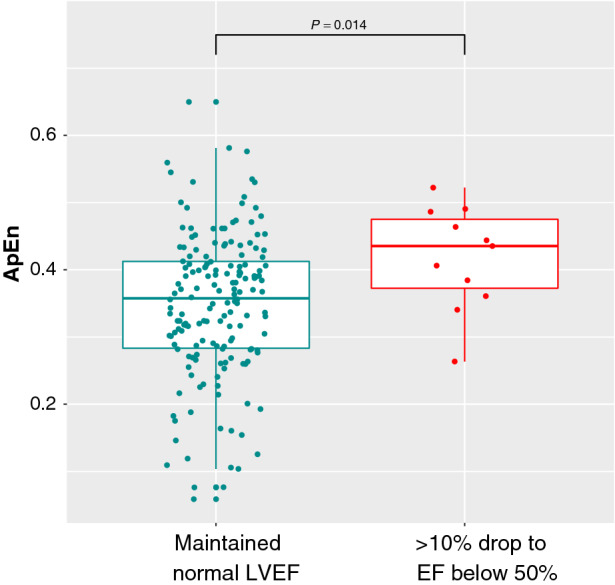
ApEn for patients calculated from baseline RNVG phase image, split into two groups based on LVEF decline

**Figure 4:**
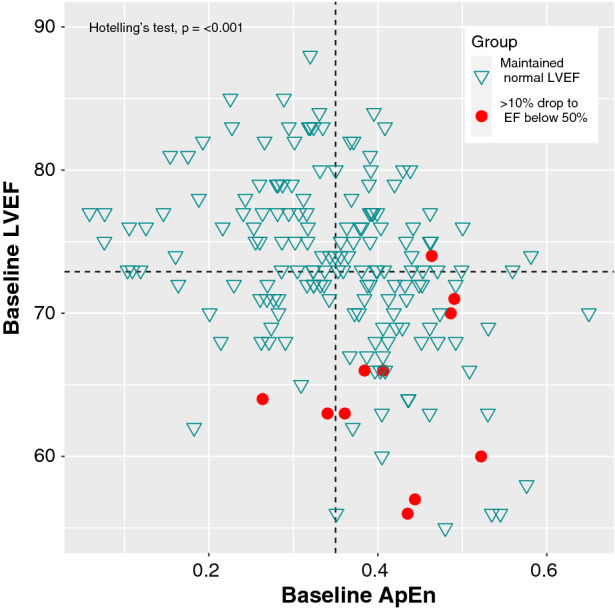
Baseline LVEF plotted against baseline ApEn for both groups. The dashed lines represent the mean ApEn and mean LVEF of the test population

A boxplot showing the results of ApEn for the groups can be seen in Figure 3. Figure 4 demonstrates how the separation between the groups can be improved by combining the baseline LVEF with the baseline ApEn. This plot indicates that patients with a lower LVEF and higher ApEn at their baseline RNVG (lower right quadrant) are more likely to have an LVEF drop of more than 10% to below 50%.

The fitted logistic regression modelling demonstrated that ApEn, baseline LVEF, and their interaction were significant predictors for CTRCD. The additional variables/predictors synchrony, entropy, and phase SD were not important when ApEn and baseline LVEF are included in the model. The AUC was 0.81 for logistic regression model 1 with all predictors and 0.88 for logistic regression model 2 with only the significant predictors and the interaction between them. A summary of the logistic regression results is shown in Table 2.

**Table 2:** Logistic regression models

Predictor	Logistic regression model 1		Logistic Regression model 2	
	Coefficient	*P* value	Coefficient	*P* value
ApEn	− 116.3147	0.044	− 99.1931	0.004
Baseline LVEF	− 0.9568	0.014	− 0.8518	0.009
ApEn, baseline LVEF interaction	1.7457	0.040	1.5253	0.033
Synchrony	241.7221	0.455		
Entropy	22.0685	0.5356		
Phase SD	0.2912	0.7761		
Model		< 0.001		< 0.001

The AUC values for the classifiers and logistic regression models are compared in Table 3. Using all predictors, an AUC of 0.87 was achieved under the Random Forest classifier and 0.78 under the Naive-Bayes classifier.

**Table 3:** Comparison of performance for each model and classifier

	AUC
Logistic regression 1 (all predictors)	0.81
Logistic regression 2 (ApEn, baseline LVEF interaction)	0.88
Random forest (all predictors)	0.87
Naive-Bayes (all predictors)	0.78

## Discussion

The results confirm that at the baseline RNVG, there was a significant difference in ApEn between the group with LVEF decline of more than 10% to below 50% (Group 2) and the group that maintained a normal LVEF throughout treatment. ApEn performed better than synchrony, entropy, and phase SD for predicting CTRCD in this dataset.

Improved discrimination between the groups was achieved by considering the combination of baseline LVEF and baseline ApEn. The results suggest that patients with a lower LVEF and higher ApEn at their baseline RNVG (lower right quadrant in Figure 4) are at a higher risk of developing CTRCD during treatment. Of the patients tested, no one who fell within the top left quadrant in Figure4 had an LVEF drop to below 50%.

The logistic regression model demonstrated the interaction between ApEn and baseline LVEF was significant between the two groups, suggesting that LVEF combined with ApEn has predictive value at the baseline scan. There was no improvement to the performance of the model when synchrony, entropy, and phase SD were included.

The classifiers performed well, achieving an AUC of 0.78 with Naive-Bayes and 0.87 with Random Forest. The Random Forest model and logistic regression model 2 performed best on this dataset. However, these results should be interpreted with caution due to the small number of patients who had a significant LVEF decline during treatment. Further work with additional data for testing would be desirable.

Published studies using echocardiography have investigated GLS to assess LV contraction abnormalities and detect sub-clinical changes before any decline in LVEF, with several studies demonstrating that a change in GLS during treatment precedes the drop in LVEF.^29^–^32^ Ali et al.^33^ found that GLS could detect subtle LV abnormalities prior to chemotherapy, and was predictive of cardiac events. They also found a significant difference in baseline LVEF between the groups. Despite there being published results using strain to demonstrate subtle abnormalities before treatment, this is the first RNVG study investigating ApEn as a predictive measure.

ApEn has shown to be promising in this patient cohort and can be calculated quickly without any additional scanning, dose, or processing time. Patients with higher ApEn and low LVEF at baseline may be more susceptible to the cardiotoxic effects of the therapy. Further improvement could potentially be achieved by combining ApEn with other clinical parameters and assessing as part of wider texture analysis. Additional data would be necessary to define a decision boundary using these parameters to highlight those most at risk.

### Limitations of Study

Of the 177 patients included in this study, only 11 patients had an LVEF decline of more than 10% to below 50%. Due to this study being retrospective, limited information was available detailing the treatment and doses, therefore this study does not discriminate between different chemotherapy regimes. Although the initial results are promising, a prospective study would be desirable to continue this work.

### Conclusions

Patients who have a normal LVEF before treatment may have subtle phase abnormalities which can be detected at the baseline test. The results of this study suggest that ApEn combined with the baseline LVEF could potentially predict which patients are at a higher risk of developing CTRCD, as measured by LVEF decline, before treatment commences. If patients who are at a higher risk of CTRCD can be identified, patient treatment and monitoring could become more personalized to the individual, helping to achieve the best outcome for each patient.

## New Knowledge Gained

Combining baseline ApEn with baseline LVEF could potentially predict which patients are at a higher risk of developing CTRCD before cardiotoxic treatment commences.

## Electronic supplementary material

Below is the link to the electronic supplementary material.

Supplementary material 1 (PPTX 302 kb)

## Abbreviations

CTRCD: Cancer therapy-related cardiac dysfunction
LVEF: Left ventricular ejection fraction
RNVG: Radionuclide ventriculography
MUGA: Multi-gated acquisition study
GLS: Global longitudinal strain
LBBB: Left bundle branch block
MI: Myocardial Infarction
ApEn: Approximate entropy
SD: Standard deviation
