# Supplementary material for: Radionuclide ventriculography phase analysis for risk stratification of patients undergoing cardiotoxic cancer therapy
Source: J Nucl Cardiol. 2020 Aug 3;29(2):581–9. doi: 10.1007/s12350-020-02277-z (PMC8993717; doi:10.1007/s12350-020-02277-z)
Supplement: Supplementary file 1 — Supplementary material 1 (PPTX 302 kb) [file 12350_2020_2277_MOESM1_ESM.pptx]

## Slide 1
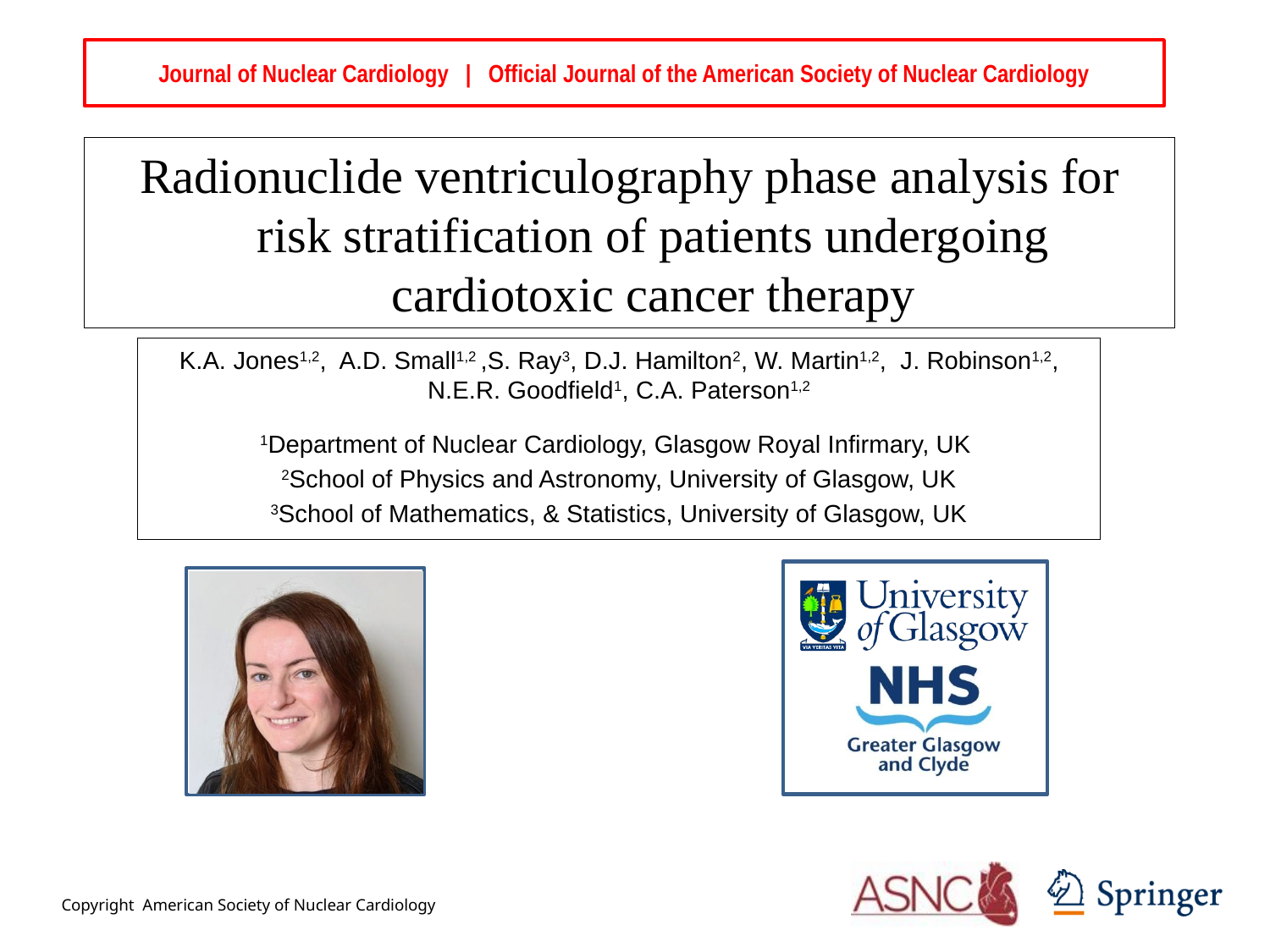

Journal of Nuclear Cardiology | Official Journal of the American Society of Nuclear Cardiology
# Radionuclide ventriculography phase analysis for risk stratification of patients undergoing cardiotoxic cancer therapy
K.A. Jones1,2, A.D. Small1,2 ,S. Ray3, D.J. Hamilton2, W. Martin1,2, J. Robinson1,2, N.E.R. Goodfield1, C.A. Paterson1,2
1Department of Nuclear Cardiology, Glasgow Royal Infirmary, UK
2School of Physics and Astronomy, University of Glasgow, UK
3School of Mathematics, & Statistics, University of Glasgow, UK
Copyright American Society of Nuclear Cardiology

## Slide 2
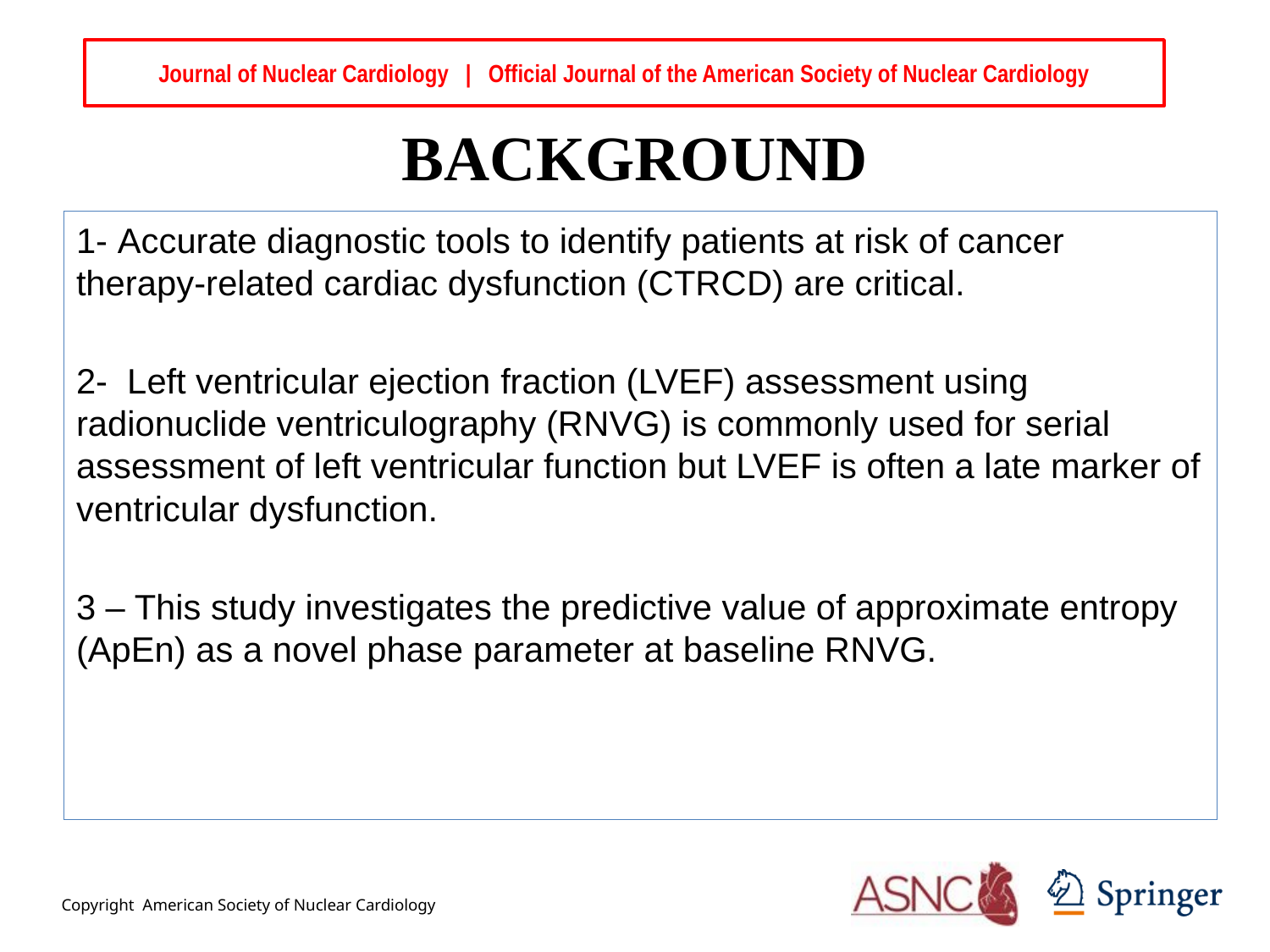

Journal of Nuclear Cardiology | Official Journal of the American Society of Nuclear Cardiology
# BACKGROUND
1- Accurate diagnostic tools to identify patients at risk of cancer therapy-related cardiac dysfunction (CTRCD) are critical.
2- Left ventricular ejection fraction (LVEF) assessment using radionuclide ventriculography (RNVG) is commonly used for serial assessment of left ventricular function but LVEF is often a late marker of ventricular dysfunction.
3 – This study investigates the predictive value of approximate entropy (ApEn) as a novel phase parameter at baseline RNVG.
Copyright American Society of Nuclear Cardiology

## Slide 3
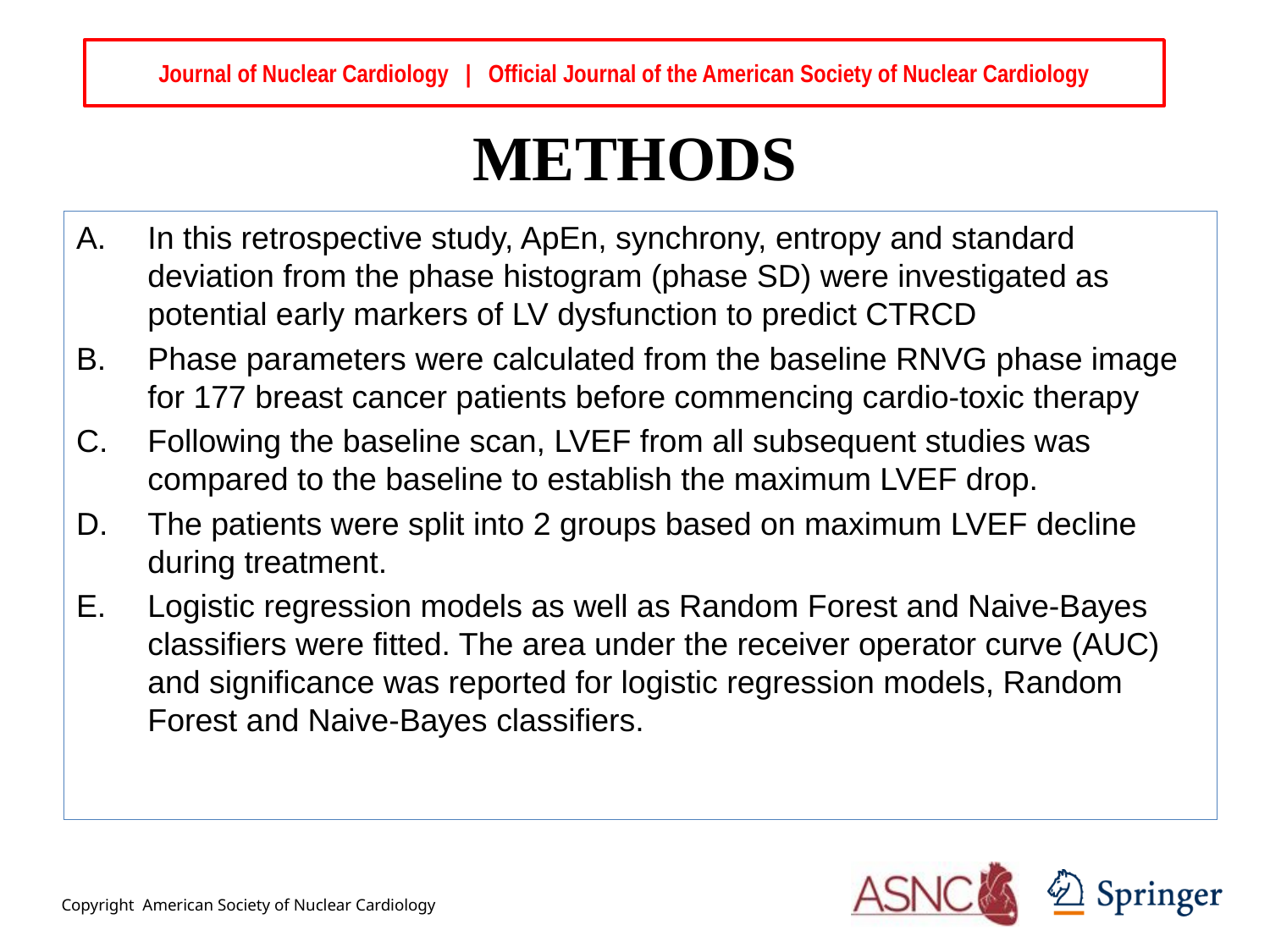

Journal of Nuclear Cardiology | Official Journal of the American Society of Nuclear Cardiology
# METHODS
In this retrospective study, ApEn, synchrony, entropy and standard deviation from the phase histogram (phase SD) were investigated as potential early markers of LV dysfunction to predict CTRCD
Phase parameters were calculated from the baseline RNVG phase image for 177 breast cancer patients before commencing cardio-toxic therapy
Following the baseline scan, LVEF from all subsequent studies was compared to the baseline to establish the maximum LVEF drop.
The patients were split into 2 groups based on maximum LVEF decline during treatment.
Logistic regression models as well as Random Forest and Naive-Bayes classifiers were fitted. The area under the receiver operator curve (AUC) and significance was reported for logistic regression models, Random Forest and Naive-Bayes classifiers.
Copyright American Society of Nuclear Cardiology

## Slide 4
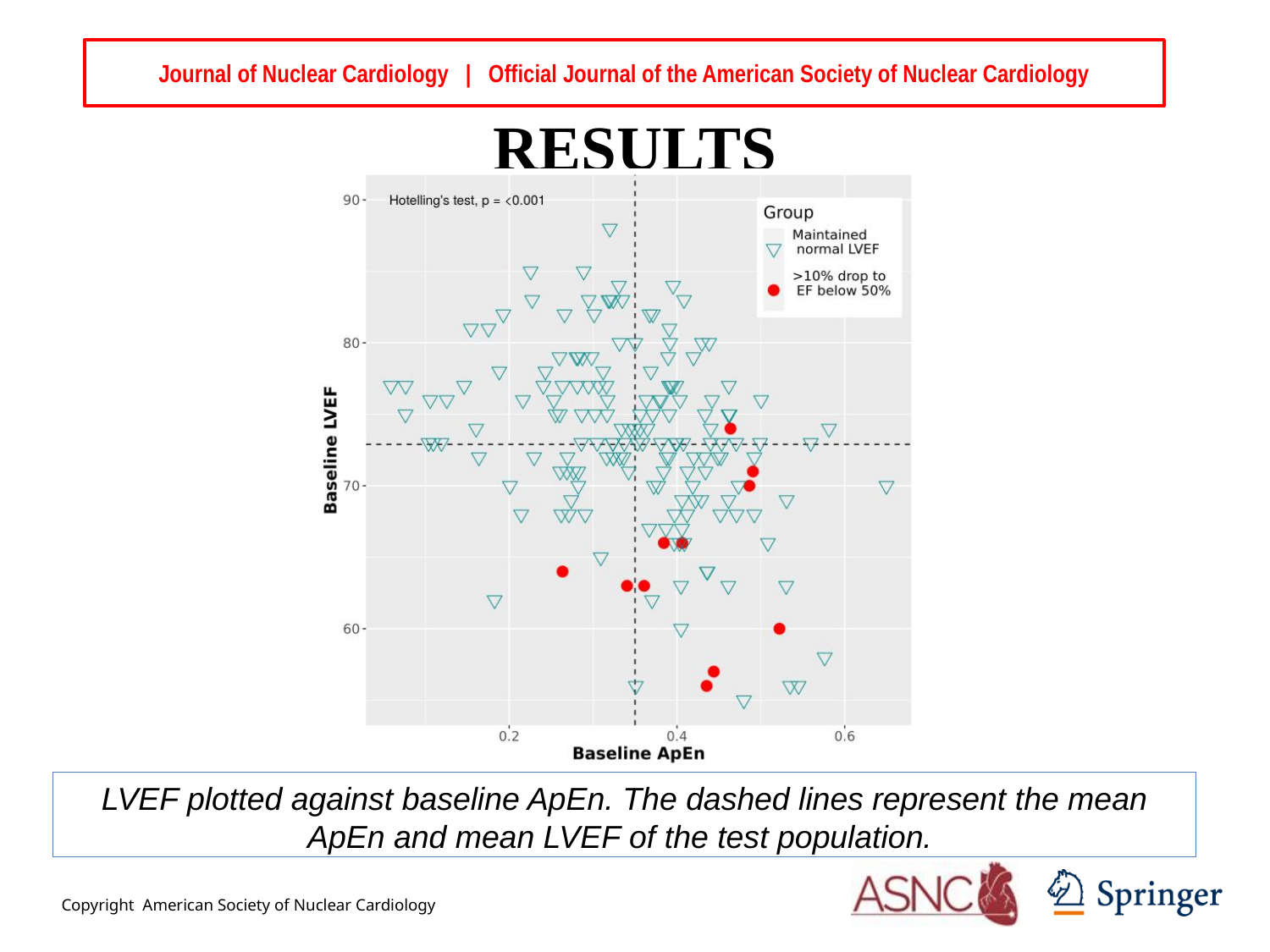

Journal of Nuclear Cardiology | Official Journal of the American Society of Nuclear Cardiology
# RESULTS
LVEF plotted against baseline ApEn. The dashed lines represent the mean ApEn and mean LVEF of the test population.
Copyright American Society of Nuclear Cardiology

## Slide 5
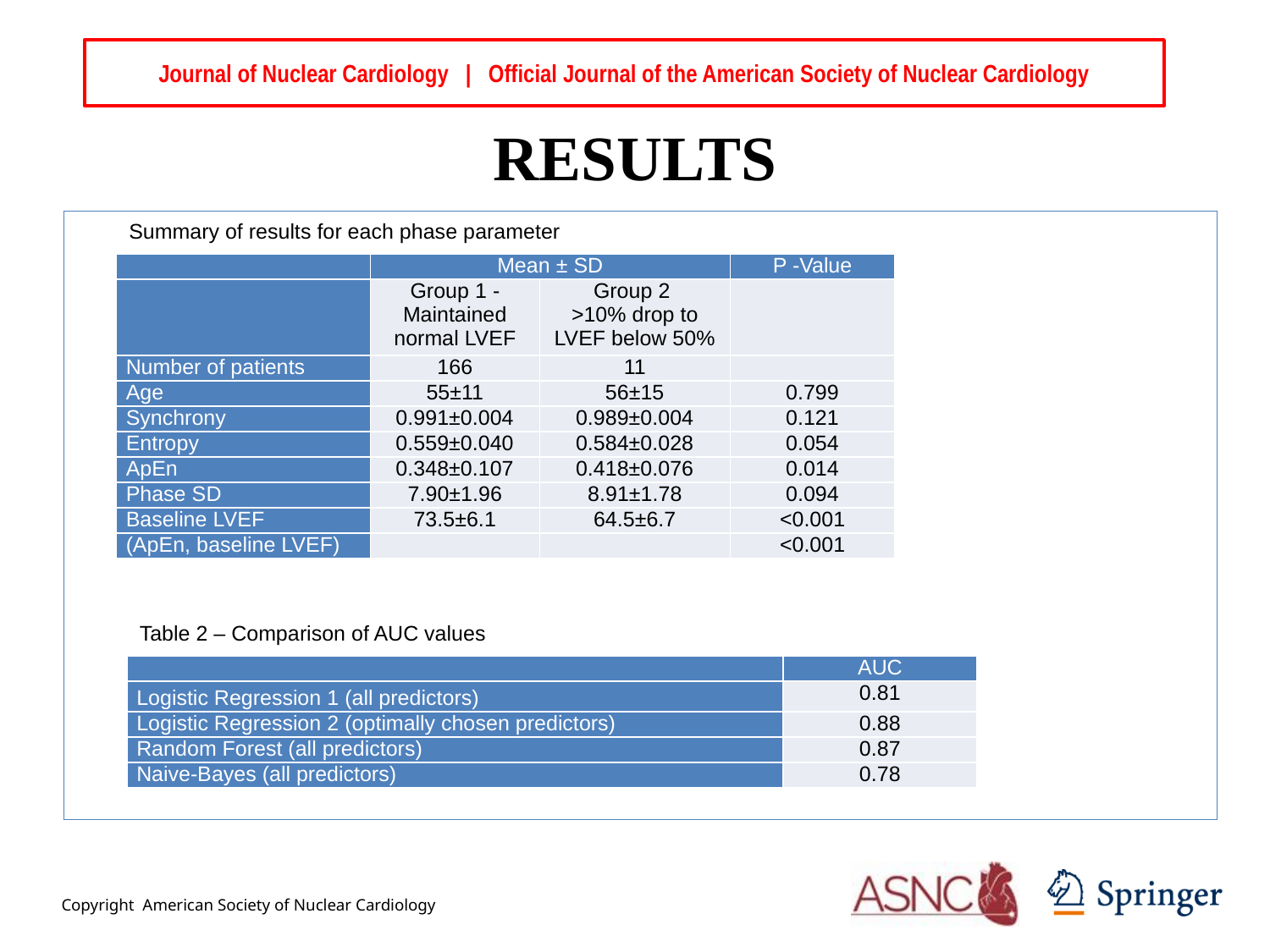

Journal of Nuclear Cardiology | Official Journal of the American Society of Nuclear Cardiology
# RESULTS
Summary of results for each phase parameter
| | Mean ± SD | | P -Value |
| --- | --- | --- | --- |
| | Group 1 - Maintained normal LVEF | Group 2 >10% drop to LVEF below 50% | |
| Number of patients | 166 | 11 | |
| Age | 55±11 | 56±15 | 0.799 |
| Synchrony | 0.991±0.004 | 0.989±0.004 | 0.121 |
| Entropy | 0.559±0.040 | 0.584±0.028 | 0.054 |
| ApEn | 0.348±0.107 | 0.418±0.076 | 0.014 |
| Phase SD | 7.90±1.96 | 8.91±1.78 | 0.094 |
| Baseline LVEF | 73.5±6.1 | 64.5±6.7 | <0.001 |
| (ApEn, baseline LVEF) | | | <0.001 |
Table 2 – Comparison of AUC values
| | AUC |
| --- | --- |
| Logistic Regression 1 (all predictors) | 0.81 |
| Logistic Regression 2 (optimally chosen predictors) | 0.88 |
| Random Forest (all predictors) | 0.87 |
| Naive-Bayes (all predictors) | 0.78 |
Copyright American Society of Nuclear Cardiology

## Slide 6
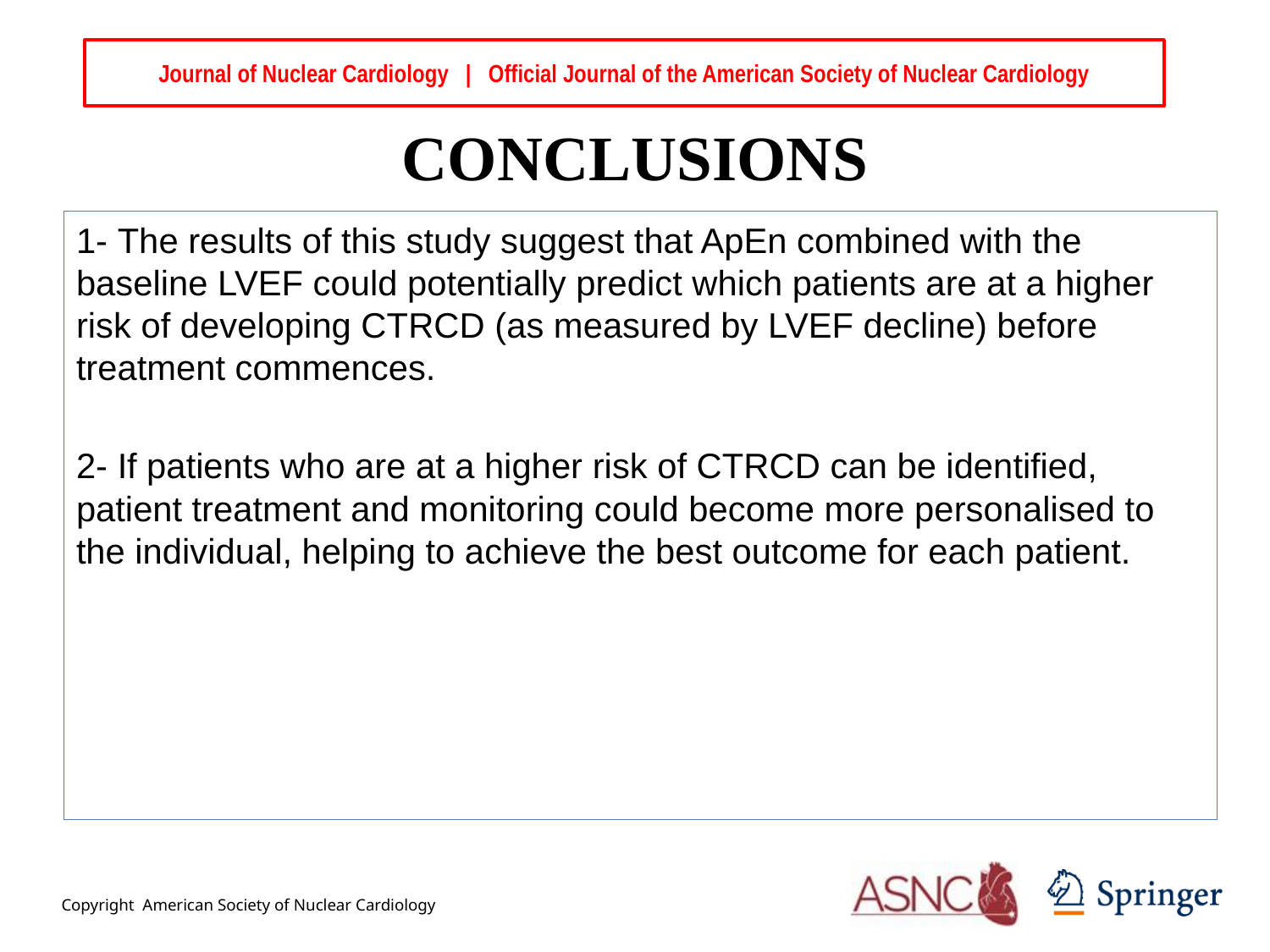

Journal of Nuclear Cardiology | Official Journal of the American Society of Nuclear Cardiology
# CONCLUSIONS
1- The results of this study suggest that ApEn combined with the baseline LVEF could potentially predict which patients are at a higher risk of developing CTRCD (as measured by LVEF decline) before treatment commences.
2- If patients who are at a higher risk of CTRCD can be identified, patient treatment and monitoring could become more personalised to the individual, helping to achieve the best outcome for each patient.
Copyright American Society of Nuclear Cardiology
